# Force and Torque Characterization in the Actuation of a Walking-Assistance, Cable-Driven Exosuit

**DOI:** 10.3390/s22114309

**Published:** 2022-06-06

**Authors:** Daniel Rodríguez Jorge, Javier Bermejo García, Ashwin Jayakumar, Rafael Lorente Moreno, Rafael Agujetas Ortiz, Francisco Romero Sánchez

**Affiliations:** 1 Departamento de Ingeniería Mecánica, Energética y de los Materiales, Escuela de Ingenierías Industriales, Universidad de Extremadura, 06006 Badajoz, Spain; javierbg@unex.es (J.B.G.); ajx2200@gmail.com (A.J.); rao@unex.es (R.A.O.); fromsan@unex.es (F.R.S.); 2 Departamento de Ortopedia, Servicio Extremeño de Salud, Hospital Universitario de Badajoz, 06006 Badajoz, Spain; rafael.lorentem@gmail.com

**Keywords:** exosuit, wearable exoskeleton, cable-driven actuation

## Abstract

Soft exosuits stand out when it comes to the development of walking-assistance devices thanks to both their higher degree of wearability, lower weight, and price compared to the bulkier equivalent rigid exoskeletons. In cable-driven exosuits, the acting force is driven by cables from the actuation system to the anchor points; thus, the user’s movement is not restricted by a rigid structure. In this paper, a 3D inverse dynamics model is proposed and integrated with a model for a cable-driven actuation to predict the required motor torque and traction force in cables for a walking-assistance exosuit during gait. Joint torques are to be shared between the user and the exosuit for different design configurations, focusing on both hip and ankle assistance. The model is expected to guide the design of the exosuit regarding aspects such as the location of the anchor points, the cable system design, and the actuation units. An inverse dynamics analysis is performed using gait kinematic data from a public dataset to predict the cable forces and position of the exosuit during gait. The obtained joint reactions and cable forces are compared with those in the literature, and prove the model to be accurate and ready to be implemented in an exosuit control scheme. The results obtained in this study are similar to those found in the literature regarding the walking study itself as well as the forces under which cables operate during gait and the cable position cycle.

## 1. Introduction

The design of walking-assistance exosuits for humans can bring noticeable benefits to those who see their walking capacities reduced due to age or pathologies. Traditional exoskeletons are rigid, bulky, and generally heavy, and are gradually giving way to wearable exoskeletons or exosuits. Even though these are relatively new to the rehabilitation field, they offer a reliable and comfortable way to increase the user’s moving capabilities without restricting movement or generating incompatibilities between the user and the exosuit’s degrees of freedom [1]. The human skeleton itself becomes the support structure. Exosuits produce force transmission to assist the human gait, and are made mostly of textile elements, drastically reducing their weight and making them much more suitable, for instance, for elderly people who see their mobility reduced. Those who need walking assistance, whether due to age or other causes (spinal cord injury or stroke, among others) are expected to significantly increase in number in both Europe and every other area with elderly population growth. By the year 2080, for instance, the population over 65 is expected to grow by roughly 30% in Europe [2]. The use of wearable exoskeletons can help such persons to improve their life quality by increasing their independence. Of late, various solutions have been reached in order to increase user mobility, through both upper limb assistance [3] and lower limb assistance [4,5]. Tendon-driven exosuits, particularly those aimed at upper-limb assistance, have been the subject of study in several recent contributions, such as [6,7,8], which examines the design of a wearable exoskeleton for lower limb assistance intended to aid gait in the elderly. As a mandatory first step in the design and control of such systems, the calculation of joint torques throughout the gait cycle must be conducted. In [9], this fact is underlined and a model for joint torque prediction is developed in the context of attending to a series of gait phases with different foot support conditions.

In order to approach the problem from a mathematical perspective, two different points of view are especially interesting, namely, inverse dynamics models [10,11] which take kinematic data as input variables, and models where the problem is directly solved based on, for example, Lagrange Mechanics [12,13]. While approaching the problem via the Lagrange Equations is less demanding in computational terms than the application of dynamic equations in multibody systems [12], inverse dynamics is an excellent method to estimate reaction forces and torques at each joint during gait without solving any differential equations. Results obtained by the Newton–Euler equations via inverse models offer a good correlation with those from multibody analysis [14]. Here, an inverse dynamics model is aimed at, assuming the gait kinematic data and the ground contact force as known quantities. Prediction of the joint torques and forces during gait is the main goal in seeking to aid the design of an exosuit for gait assistance. A similar inverse dynamics model was presented in [10] for the whole body. Kinematic data such as position, acceleration, and segment angles can be measured using inertial measurement units and/or markers with a camera setup, as in [2], while ground contact forces can be measured via sensors embedded in the shoes, as presented in [15]. While there exist certain flexible exoskeleton proposals to assist the elderly, as in [16], soft exosuits with no support structure, as proposed in [17], provide the best case scenario for near-zero suit–human interaction.

Inverse dynamic models have been extensively used when designing exosuits for upper limb assistance, as in [18,19,20]. Inverse dynamics in the field of lower-limb assistive exosuits can be found in the recent literature, as in [2], although the results have rarely been studied with regard to their implication on the design of an exosuit. A general cable model for hip and ankle assistance during gait is presented in this paper, including the human body and cable stiffness effects. These two joints were chosen because they are the main power contributors during gait, as per [4,21]. A single-cable configuration is followed in this study, that is, torque can be transmitted in only one direction and the cable follows a certain trajectory along the human body via a set of pulleys or general cable guides. Multiple trajectory points along the path from the actuation system to the anchor points are considered in order to reliably predict the cable extension at both joints. Bowden cables could be used for the design of the exosuit, with the consequent non-negligible efficiency loss due to non-linear friction between the cable and the sleeve [22,23]. Because the model is a fully 3D approach to human motion inverse dynamics, it is possible to study the effects of the action of flexion–extension torque at a certain joint upon its abd/adduction and rotation torques, as well as to predict the cable’s instant position regardless of the pulley configuration. Avoiding excessive undesired joint torques outside the sagittal plane may be a key design factor when approaching the positioning of the anchor points or the transmission system, among others; the model presented here can be used to parametrically quantify these torques as functions of said design variables. As the actuation depends on the results directly provided by the inverse dynamics model, the influence of gait and anthropometric data for a certain subject on the design or operation of the exosuit can be quantified as well. Here, the predicted torque at the motor shaft and the cable extension during gait are studied for certain gait conditions, namely, those of an elderly subject walking overground at a comfortable speed.

## 2. Materials and Methods

### 2.1. An Inverse Dynamics Model for Human Gait

In this section, the modeling of dynamic equations leading up to the derivation of joint torques and reaction forces is presented. Gait kinematic data, along with the ground contact forces, are input data. Applying Equations (1) and (2) with respect to a fixed inertial frame of reference will lead to the desired expressions. Such equations are applied as follows to each j-th leg segment and for each of the three global reference axes:

(1) $$\sum F_{j}=m_{j}a_{j}$$

(2) $$\sum n_{j}=I_{j}{\dot{\omega }}_{j}+\omega_{j}\times \left(I_{j}\omega_{j}\right)$$

In (1) and (2), $F_{j}$ stands for the force on segment *j*, $a_{j}$ for the linear acceleration of the center of mass of segment *j*, $m_{j}$ for its mass, and $I_{j}$ for its inertia tensor, while $\omega_{j}$ is the angular velocity of segment *j*.

**Figure 1:**
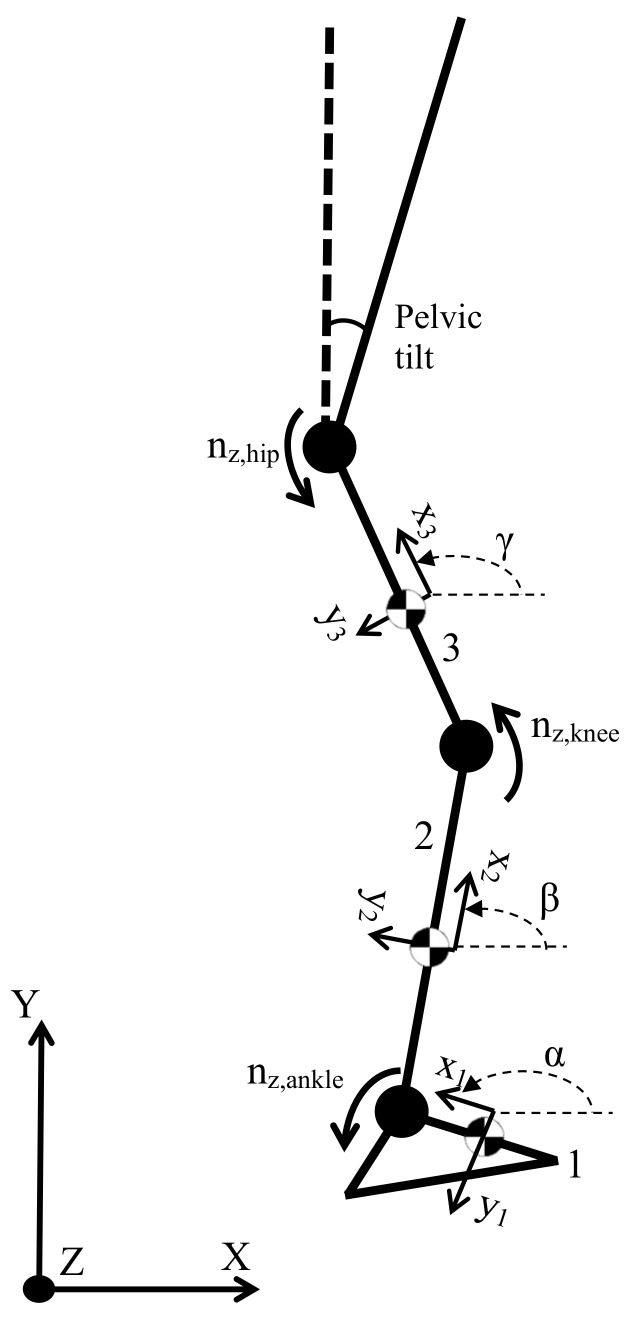
Simplified scheme for the considered lower-limb segments.

As the main goal is the deduction of torques and forces in the ankle, knee, and hip, a simple three-dimensional model for the leg is chosen. The full system consists of three segments plus the torso, all joined to one another, and each of them solitary to its own local reference frame. Joints allow rotational displacement around the three coordinate axes; thus, the linear reaction forces and the joint torques are the unknowns to be solved. Figure 1 shows the most essential elements in such a model, omitting for better clarity joint forces and torques other than those in the z-axis. Such local reference frames are defined by their corresponding Euler angles in the common Z-Y-X order.

For each leg, Equations (1) and (2) are a system of eighteen differential equations: nine for force, nine for torque, and six in total for each leg segment. Said equations can be transformed into algebraic equations with the position data for each point (the centers of gravity of each leg segment and their joints), segment angles, and linear and angular accelerations as known quantities, which can be obtained by gait analysis in the laboratory. Equations (1) and (2), as particularized for each leg segment, are therefore as follows.

- Segment 1, foot:
  (3) $$F_{a,i}=m_{1}\left(a_{1,i}-g_{i}\right)-F_{r,i}\;\;\;\;\;i=x,y,z$$
  (4) $$n_{a,i}=I_{1,i}{\dot{\omega }}_{1,i}+{\left.\left(\omega_{1}\times \left(I_{1}\omega_{1}\right)\right)\right|}_{i}-{\left.\left(r_{ga}^{P}\times F_{r}\right)\right|}_{i}-{\left.\left(r_{ga}^{A}\times F_{a}\right)\right|}_{i}$$
  In (4), ${\dot{\omega }}_{1i}$ represents the angular acceleration of the foot segment around its center of gravity with respect to the fixed *i* axis of the global reference frame, while $\theta_{ji}$ is the Euler angle *i* of segment *j*. The ground contact force is referred to as $F_{r,i}$ and the force and torque at joint *j* (a for ankle, k for knee, and h for hip) are $F_{j,i}$ and $n_{j,i}$, respectively. COM stands for the center of mass and vectors are described such that their subindex is their origin; $r_{ga}^{P}$, for instance, is a vector from the foot’s COM to the application point of the ground contact force.
- Segment 2, shank or leg lower segment:
  (5) $$F_{k,i}=m_{2}\left(a_{2,i}-g_{i}\right)+F_{a,i}\;\;\;\;\;i=x,y,z$$
  (6) $$n_{k,i}=I_{2,i}{\dot{\omega }}_{2,i}+{\left.\left(\omega_{2}\times \left(I_{2}\omega_{2}\right)\right)\right|}_{i}-{\left.\left(r_{gk}^{k}\times F_{k}\right)\right|}_{i}+{\left.\left(r_{gk}^{A}\times F_{a}\right)\right|}_{i}+n_{a,i}$$
  The force $F_{a,i}$ calculated in (3) was the joint force exerted by the rest of the body upon the foot segment. In (5) and (6), however, the same $F_{a,i}$ stands for the force exerted by the foot upon the shank segment; thus, the sign changes. The same applies to the moment, $n_{a,i}$ in (6).
- Segment 3, thigh or leg upper segment:
  (7) $$F_{h,i}=m_{3}\left(a_{3,i}-g_{i}\right)+F_{k,i}\;\;\;\;\;i=x,y,z$$
  (8) $$n_{h,i}=I_{3,i}{\dot{\omega }}_{3,i}+{\left.\left(\omega_{3}\times \left(I_{3}\omega_{3}\right)\right)\right|}_{i}-{\left.\left(r_{gh}^{h}\times F_{r}\right)\right|}_{i}+{\left.\left(r_{gh}^{k}\times F_{k}\right)\right|}_{i}+n_{k,i}$$

Combining all of the previous equations, all of them referring to the inertial frame shown in Figure 1, provides the system of equations to be solved.

### 2.2. Approach to the Design of a Lower-Limb Assistance Exosuit

**Figure 2:**
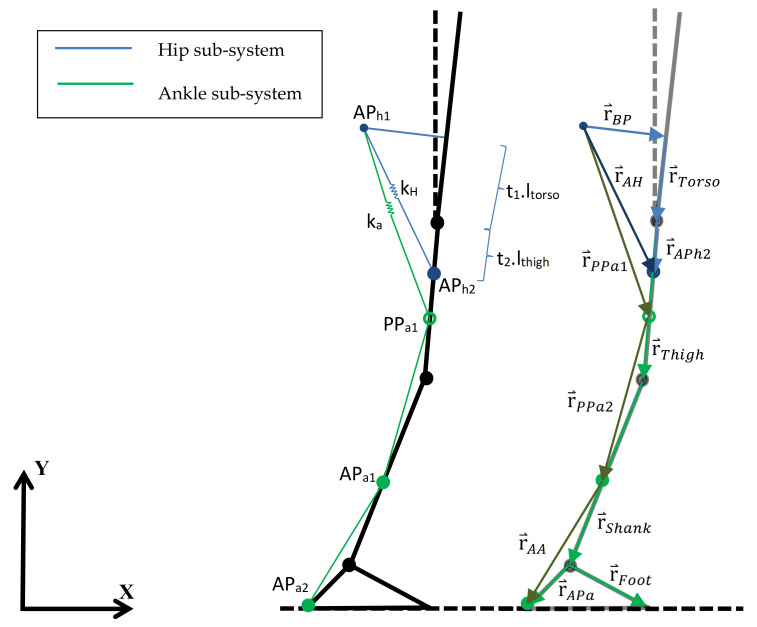
Geometric model for calculating the cable traction forces from the expected joint torques in both the hip and ankle sub-systems.

When the joint torques and reaction forces are known for each time point during gait for the given kinematic data, it is possible to use said information to, for instance, control the action of the exosuit’s actuators, which then use cables to produce a determined percentage of the total required joint torque, thus assisting the gait of the wearer. Next, a general geometric model is introduced to translate this percentage of the joint torque applied in the corresponding joint into the required forces in the cables, and consequently into the torque curve for the actuators. In this work, we particularize the general scheme to an exosuit that assists hip and ankle joints as an application example of the proposed model. A scheme for the hip and ankle joints is shown in Figure 2.

Generally, a more simplified geometric model is developed to obtain the relation between cable forces and joint applied torques, as in [18], where the problem is restricted to a 2D cable-joint interaction; although this simplifies the problem, it is missing out-of-plane information. For this multiarticular assistive exosuit, however, a precise geometric model is required in order to properly predict cable forces based on the desired instant torque as well as to provide a reliable model for the cables’ extension. Therefore, a generalization of the method developed in [5] is proposed in this work, using the biomechanical model of the subject defined in the previous section in combination with an exosuit. In [5], a simplified 2D model for the hip actuation is presented, adding the human body’s stiffness; however, the the cable’s stiffness is neglected, and out-of-plane motion is ignored because the proposed exosuit is not expected to act under either ab- or adduction. However, cable misalignment might lead to undesired out-of-plane torques only approachable with a 3D cable model. Here, as both hip and ankle are actuated, the exosuit has two actuation units (one per joint) acting alternatively on both sides. Thus, the model considers two different subsystems for the actuation, one to assist the hip joint (Figure 2, blue) and the other to assist the ankle joint (Figure 2, green). The pulleys driving the cables are located at a certain distance perpendicular to the torso corresponding to the backpack where the exosuit actuation system is located. While the cable belonging to the hip subsystem goes straight to the anchor point, the one in the ankle sub-system follows a certain path defined by a number of trajectory points and the two final anchor points. The cable passes through such trajectory points at any given instant during gait, as in cable guides. The desired flexion/extension torque at any specific instant is known from the inverse analysis, while the existing relationship between such torque and the required cable force to achieve it is shown below:

(9) $$n_{mj,z}=-r_{APj}\times {\left.f\right|}_{z}$$

In (9), $n_{mj}$ stands for the motor torque applied to joint *j* and f for the cable force, while $r_{APj}$ is a vector joining the joint *j* to the final anchor point.

The desired motor torque in joint *j* is generally expressed as a percentage of the corresponding biological joint torque whenever the consequent cable force is traction. The minus sign allows positive values to be obtained for the actuation range, where the desired torque is negative (clockwise). Constants $k_{i}$ in Figure 2 represent the total equivalent stiffness of each sub-system, that is, the combined effect of the cable stiffness and that of the human body in each case. The latter can be approached via experimental methods, such as those described in [5], where the body responds to any force following a parabolic law. Because every exosuit defining vector shown in Figure 2 is strictly related to the body motion itself, their kinematics being completely defined, their coordinates and modulus are known along the gait cycle.

When the required joint torque is known and the desired percentage of additional support by the actuation system in each gait stage is defined, the necessary force in each cable can be directly obtained from (9). In cases where the obtained value for $n_{h3,z}$ is negative in certain periods, the cable is not able to actuate, as it cannot, in general, produce compression; the results will therefore be zero for said stages. If the reduction ratio of the gearbox installed with the actuators is known, along with the pulley array that transmits the movement, it is possible to obtain the instantaneous torque that must be demanded to the actuation system throughout gait. Furthermore, the total cable extension for each sub-system is known by considering both the inverse dynamics results and the cable force to predict the cable extension due to cable and body stiffness.

## 3. Results and Discussion

### 3.1. Inverse Dynamics Results

In order to check the model’s reliability, databases as in [24] can be accessed and used to obtain the instantaneous values for joint torques during the walking cycle for a series of studied cases at different gait speeds. There, the authors provide an extensive set of kinematic results for a large number of subjects while walking overground or on a treadmill using camera-based tracking. Additionally, as in [24], with numerical data for the kinematic and ground contact force parameters for each time during gait the full solution can be reached via the proposed method. That is, simply by introducing the input parameters, the corresponding force and torque reaction values in the three axes at each joint can be obtained.

Information regarding the geometric position for the center of gravity of each considered leg segment for each leg can be easily calculated from the joint position data collected in [24]. Ground contact force is included in [24] for each of the three coordinate axes as well, along with the position of its center of pressure. As the data published in [24] correspond to a statistical analysis conducted for several cases, average anthropometric data according to [25] have been used with the proposed model. In [25], the average segment length and mass are normalized by the subject’s height and total mass, as commonly used in applied software when testing several different subjects. In order to fully test the model’s reliability on a target population, a group of ten subjects from [24] was selected, with ages ranging from 50 to 73 and both male and female, while walking over ground at comfortable speed. In [24], said subjects are referred to as subjects 27, 28, 29, 31, 33, 34, 35, 37, 41 and 42. Average anthropometric data as functions of each subject’s height and mass are used, based on the common model described in [26], including segment length and mass, moments of inertia, etcetera; [26] provides a model for the lower limb specifically that is commonly used in numerical software for gait and human movement analysis. All necessary kinematic and ground contact force information is filtered using a fourth-order low-pass Butterworth filter using a cut-off frequency of 10 Hz, with sample rates of 150 Hz (kinematic) and 300 Hz (ground contact force and its center of pressure, COP), as indicated in [24]. Such a filter design is broadly used in the biomechanics field, including the authors of the dataset used in [27], where the authors provide another dataset focusing on running subjects instead of normal walking.

**Figure 3:**
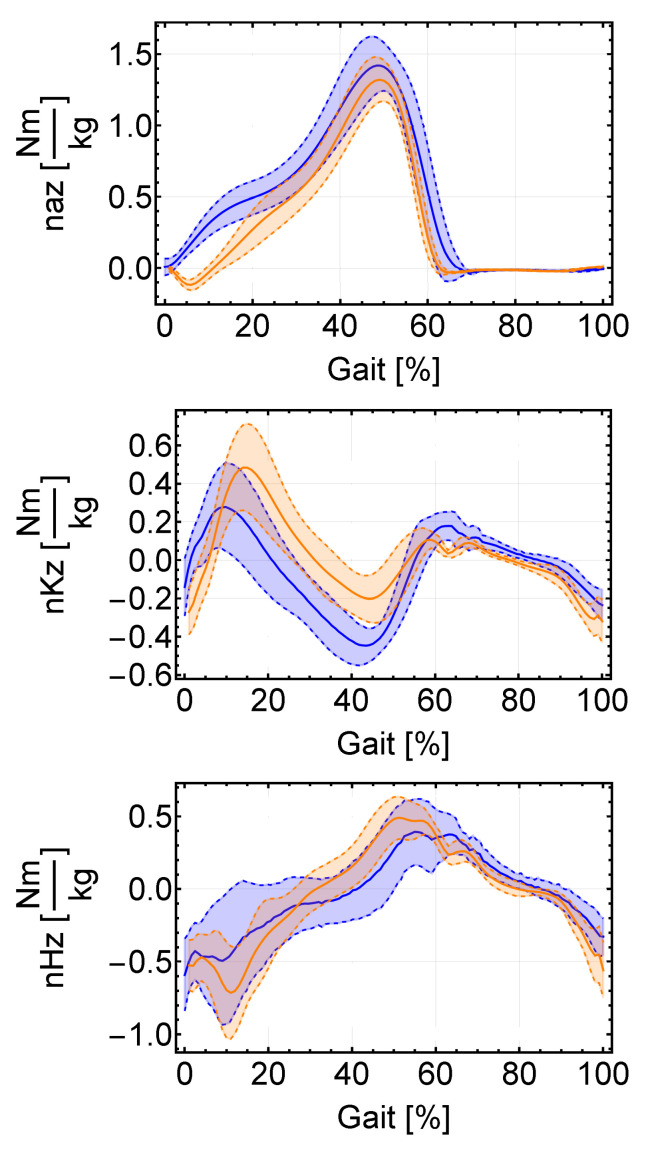
Representation of the normalized joint torques obtained via the proposed model for the selected population, according to anthropometric data in [25] (blue) and those in [24] for the “comfortable” gait speed (orange). Average values as solid lines. In dotted lines, ±standard deviation.

Using the anthropometric data as input for the model along with the kinematic variables in [24], the problem is already completely defined for its solution via the proposed dynamic inverse model. A system of eighteen equations with eighteen unknowns remains to be solved. Figure 3 shows the results for flexion–extension torques at the ankle, knee, and hip joints calculated in [24] and those obtained for the selected population, showing the average results and the standard deviation. Similar results to those included in [24] are obtained, although subject to deviations due mainly to the use of average anthropometric data in [25], such as segment moments of inertia or position of their centers of gravity. Anthropometric data are very relevant to the final solution, and information such as the exact position of the ground force center of pressure greatly impacts the result; the exact position of the COP during the contact phase is very determinant and a possible source of errors, showing in Figure 3 a stronger deviation in that phase. Moments in the upper joints are more sensitive to the solution of the lower segments, and might require different filtering in order to yield more accurate results, as stated in [28] wuth respect to the results of a large study in low-pass filtering focusing on inverse dynamics.

In [24], several attempts were performed for each subject, while only the average inverse kinematics results are provided. Because subjects were selected while walking over the ground at different speeds, comparing the average results with those of an specific attempt might be a considerable source of error. However, this way was chosen in order to analyze the problem from a realistic perspective; when designing an exosuit for walking assistance, the required actuation strongly depends on the walking conditions. Thus, a treadmill at constant speed was rejected as a means of studying variable phenomenon. In addition, the results are very different between subjects, as their height ranges from less than 1.5 m to more than 1.8.

### 3.2. Motor Torque in a Wearable Exosuit

Knowing the joint torque patterns during gait is especially interesting regarding the development of a control scheme for the walking-assistance exosuit actuation system, the calculation of the forces applied to the tires, and the demanded motor torque. In order to estimate those forces and torques for the selected population using the data in [25] (Table 1), Equation (9) can be applied to reach an approximation for the case in Figure 2. Thus, a demanded joint torque of 30% of the total at the hip joint is established, as in [5], and 15% at the ankle joint, in order not to go beyond the motor maximum continuous torque. An actuation system characterized by the parameters in Table 1 can now be designed.

In Table 1, $t_{1}$ and $t_{2}$ represent, for the hip-subsystem, the distance from the contact point between the exosuit backpack and the back and the hip joint ($t_{1}$) and between the hip joint and the hip subsystem anchor point ($t_{2}$) as fractions of the torso and thigh segment lengths, respectively (as in Figure 2). They are similarly defined for the ankle subsystem. The path followed by the ankle cable is defined by two trajectory points located, respectively, at 80% of the thigh and 30% of the shank segment. These positions are chosen in order to locate the first trajectory point at a distant position from the final anchor point in the hip-subsystem, as well as to improve the scheme’s visibility. How many trajectory points are included and where they are located impacts the cable extension function; these are therefore relevant factors in the design of the exosuit. In this case, the ankle anchor point is located at the same height as the heel camera-based marker except with the same y coordinate as the ankle to induce as little abduction moment as possible. Human body stiffness is neglected in the first place, although it is taken into account later on, and the cable stiffness is assigned a value of 6 × 10${}^{5}$ N/m, following [5]. Now, the cable forces can be obtained and compared to those in [5] for the hip subsystem. To calculate the required cable force during gait, the biological hip moment in Figure 3 is filtered with a 10 Hz low-pass filter to avoid sudden changes in the moment being demanded of the engine, increasing user comfort [5].

**Figure 4:**
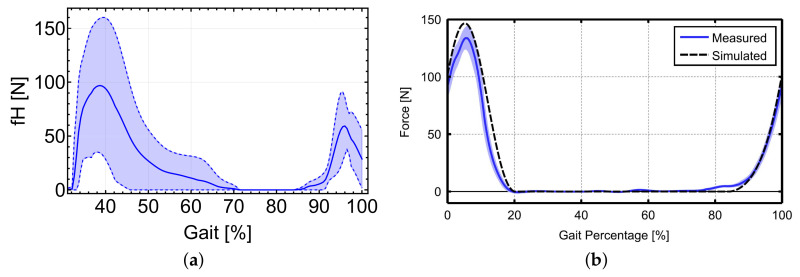
(**a**) Cable force obtained using the proposed model for the data in [24] to assist 30% of the total torque. Average values as solid lines. In dotted lines, ±standard deviation. (**b**) Representation of the cable force of the hip subsystem according to [5].

**Figure 5:**
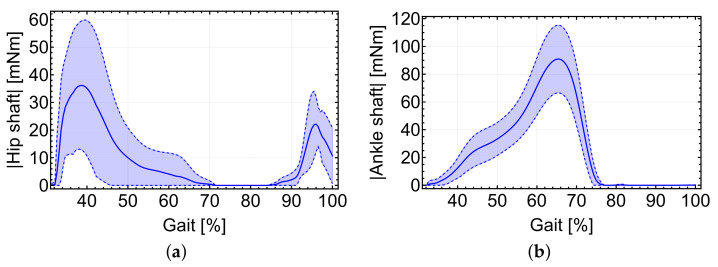
(**a**) Torque at the motor shaft during the walking cycle for the hip subsystem and (**b**) for the ankle sub-system. Average values as solid lines. In dotted lines, ±standard deviation.

Rather than comparing the exact values obtained in both cases for the cable force, Figure 4 is interesting in the way it reveals how the evolution of force varies in a similar trend for both. Individuals tested for each method had different biological characteristics, and therefore the results are not directly comparable. Moreover, the biological hip moment used in [5] was smoothed differently. Instantaneous torque demanded at the motor shaft can be predicted, which can assist 30% of the hip and 15% of the ankle joint torques during gait for both hip and ankle joints, as shown in Figure 5.

It is notable that in the case of the hip actuation there exists a walking phase, between 40% and 85% of the gait full cycle approximately, where both the force in the cable and the required actuation torque might be zero (Figure 4 and Figure 5). In all cases, the gait cycle shown in the figures starts at 0% when the right heel first touches the ground and finishes at 100% when the same heel hits the ground at the start of the next cycle. During this phase, if the cable were to produce work it would be in the form of compression, which it is incapable of doing in general, and the same applies to the ankle. On the other hand, intense use of the actuators is predicted in the heel support phase and a portion of the full plantar support phase (up to around 65% of the gait cycle), as well as in the final stage of the swing phase (from around 65% of the gait cycle onwards) for the case of the hip subsystem. The maximum torque is required at the end of the heel support phase. Additionally, the peak in required torque at the ankle happens during the full foot support phase.

**Figure 6:**
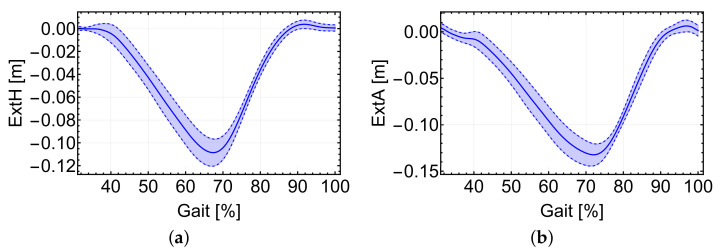
(**a**) Cable extension during the walking cycle for the hip and (**b**) ankle subsystems. Average values as solid lines. In dotted lines, ±standard deviation.

The total cable extension for each system is the first step for the motor position control. As the cable vectors are fully defined, it is possible to obtain the total cable extension with respect to the initial length (that at the beginning of the gait cycle). Figure 6 shows the extension of both hip and ankle cables without considering any cable or body stiffnesses. Increasing or decreasing the number and/or location of the trajectory points in the ankle subsystem highly modifies the instantaneous cable extension.

Looking at the two graphs, a maximum decrease in cable length of approximately 12 cm and 15 cm, respectively, can be seen for each subsystems. Increasing the number of trajectory points for the ankle subsystem modifies the cable extension and is therefore relevant for implementing the position-based control scheme. The more cables in both systems deviate from being in-plane with the trajectory and anchor points, the higher a force needs to be transmitted by the cable to aid the same percentage of joint torque. Additional torques in x and y appear as well, which can be calculated when the required cable force is known, and can affect the user comfort.

**Figure 7:**
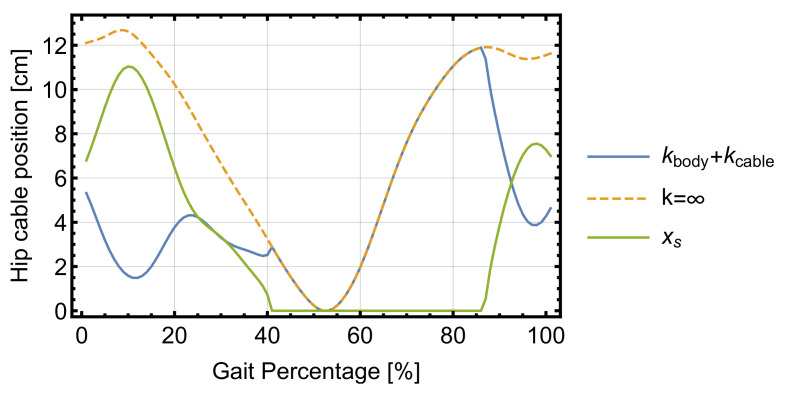
Cable position at the motor with and without stiffness along with $x_{s}$.

**Figure 8:**
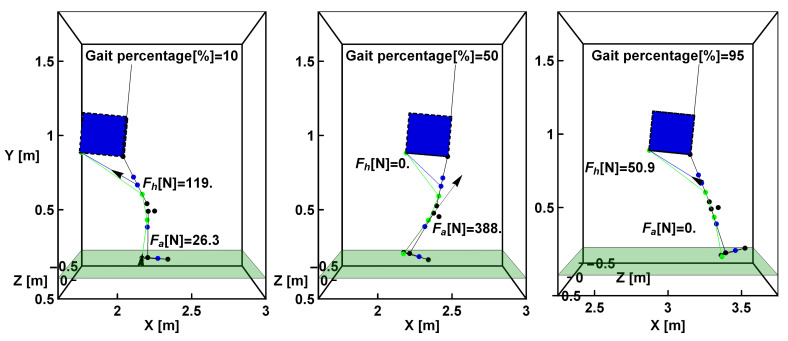
3D model of the exosuit along with the lower segment and torso.

Cable and human body stiffnesses have a great impact when controlling the actual cable position at the motor–pulley system. As an example, both kinds of stiffness data presented in [5] for the hip sub-system are used with the proposed model to obtain the instantaneous cable position at the pulley and the total cable plus human body elongation for subject 29 in [24]. An average cable stiffness of 6 × 10${}^{6}$ N/m is considered for the cable, while a non-linear stiffness model is assumed for the human body, defined in [5] as:

(10) $$\begin{array}{c} F\left(x_{s}\right)=8899.7x_{s}^{2}+99.546x_{s} \end{array}$$

where $x_{s}$ is defined in [5] as the total length of the equivalent spring measured in meters and F is the cable force at the hip sub-system measured in N. The spring stands for both the cable and human stiffness, the latter including the compression in thigh, waist, and shoulder (where the exosuit’s backpack is in contact with the human body). Hip cable extension can be obtained by considering the stiffness effect, calculating only the elongation due to the instantaneous cable force, which is already a known quantity. Thus, Figure 7 shows the cable extension with and without the stiffness effects, the latter being the one shown in Figure 6a except with the zero located at the minimum length and the equivalent spring length $x_{s}$:

While the cable stiffness has a negligible effect due to its high value, it would probably be relevant if Bowden cables were used, as friction losses have a notable effect on their performance. However, the human body stiffness produces a displacement that reaches values of up to 11 cm in the studied case. Figure 8 shows the 3D model for the exosuit and the required cable force for 10%, 50%, and 95% of the gait cycle, showing the exosuit’s backpack, the cables for both systems, and their corresponding trajectory and anchor points. All measurements correspond to the case solved in this section, without considering the body stiffness for either the hip or ankle joints.

Both systems act simultaneously at various stages during gait, although their peak values are reached at different times, as shown in Figure 4. The maximum value of the ankle cable force is noticeably higher than that of the hip, although this strongly depends on the exact position of the anchor point. The higher the distance from the ankle, the lower the force required to assist the same torque.

## 4. Conclusions

Here, an inverse dynamics model has been proposed for the analysis of joint reaction forces and torques during gait. Said model takes into consideration six degrees of freedom for each leg segment (three linear and three rotational) and takes the ground reaction force and the walking kinematic parameters of linear and angular position and acceleration as input variables. Flexion torques in hip, knee, and ankle were calculated and compared with those found in the literature as a preliminary step for the design of a walking-assistance exosuit. As the model is defined from the Euler angles, it is a full 3D inverse dynamics model and can be used as such as long as enough markers are used to determine each segment’s angles, making possible an analysis of the impact of several anthropometric parameters on walking dynamics and their impact on the exosuit’s design.

As a first step towards the design of a soft wearable exoskeleton for walking assistance, the capacity to fully predict the required joint torque to assist the user during gait is mandatory. The proposed model has been tested using bibliography data. Following installation in the exosuit along with the corresponding control scheme, it must be capable of receiving such information from the sensors and requesting the correct torque from the actuation system at each instance. As an implementation of a generally-designed exosuit, a 3D model for cable-driven actuation has been chosen to precisely predict the required motor torque and traction force in cables for the walking cycle. Outcomes close to those in the literature were obtained via the proposed inverse model along with a torque, force, and cable extension for both the hip and ankle actuation. The results obtained were acceptable regarding both the walking study itself (reaction forces and torques from the input ground force and kinematic data) and the forces under which cables can be expected to operate during gait. The described mathematical model is expected to be implemented in the control scheme for the currently under-development exosuit. It will be integrated using a musculoskeletal model to optimize coordination between the exosuit’s actuation and the muscular system, allowing it to determine the best way to actuate when a certain muscle is handicapped.

## Figures and Tables

**Table 1:** General characteristics for the actuation system in a wearable exoskeleton.

	Hip Sub-System	Ankle Sub-System
Max. continuous torque [Nm]	4	4
Max. continuous torque at the motor shaft [mNm]	95.6	95.6
Transmission ratio [-]	1:33	1:79
Pulley radius [m]	0.0126	0.019
*t*_1_ [-]	0	0
*t*_2_ [-]	0.6	0.8
*t*_3_ [-]	-	0.3
Backpack length [m]	0.32	0.32
Trajectory points	0	2

## Data Availability

The data that support the findings of this study are openly available in [24] at https://pubmed.ncbi.nlm.nih.gov/29707431/ (accessed on 18 April 2022).
